# Supplementary material for: Structure of cyanobacterial photosystem I complexed with ferredoxin at 1.97 Å resolution
Source: Commun Biol. 2022 Sep 12;5:951. doi: 10.1038/s42003-022-03926-4 (PMC9467995; doi:10.1038/s42003-022-03926-4)
Supplement: Supplementary file 2 — Supplementary Information [file 42003_2022_3926_MOESM2_ESM.pdf]

Supplementary information

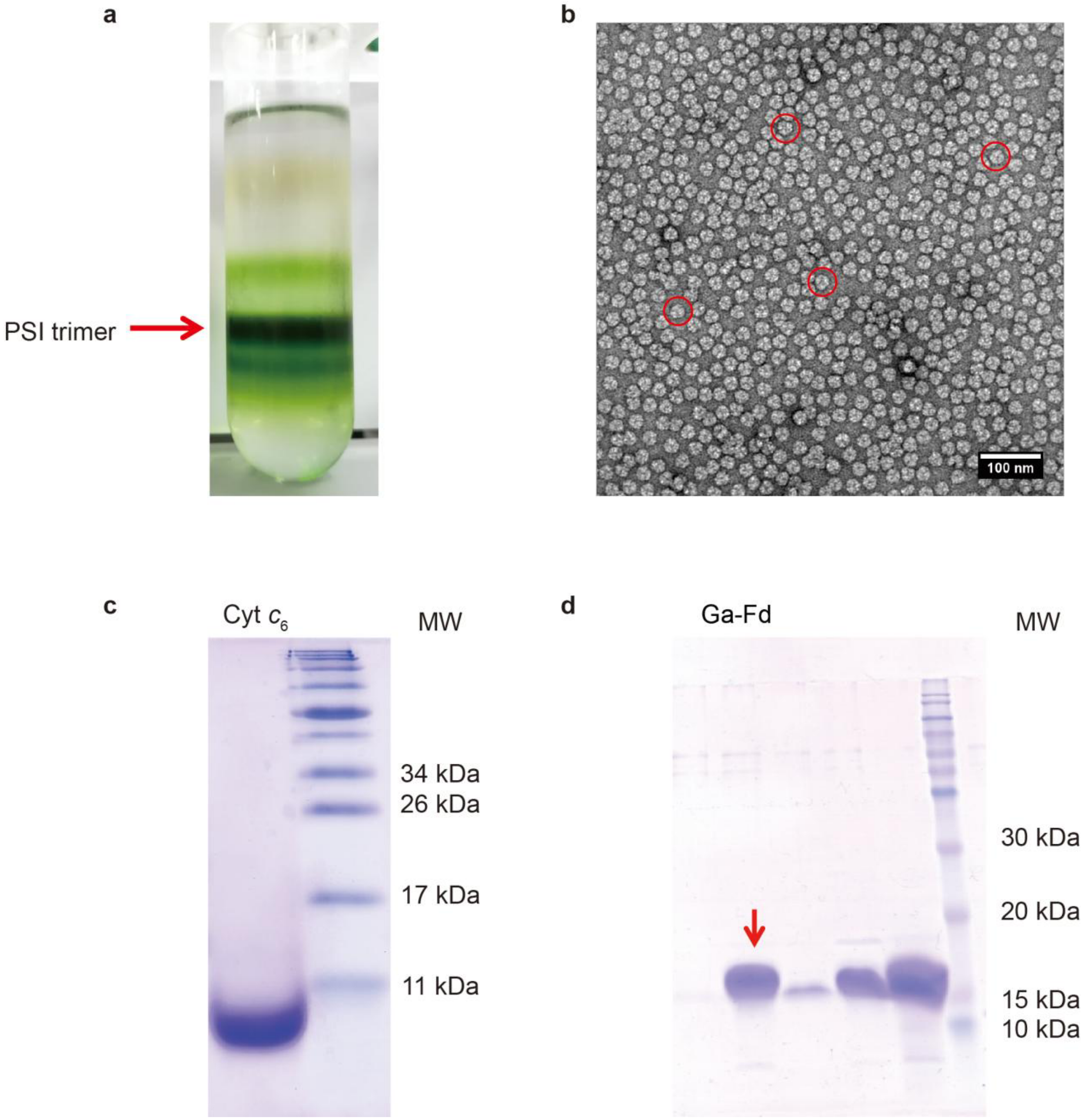

Supplementary Figure 1. Purification of the PSI trimer, Ga-Fd and Cyt c_6.

a Sucrose density gradient centrifugation of PSI after Ni-NTA affinity chromatography. The band corresponding to PSI trimers is highlighted by a red arrow.

b Micrograph of a negatively stained PSI trimer preparation. Representative PSI trimer particles with easy to discern protomers in the top-view are circled in red.

c SDS-PAGE of purified native Cyt c_6.

d SDS-PAGE of purified Ga-substituted Ferredoxin (Ga-Fd). The Coomassie blue stained band corresponding to the final purification step is marked by a red arrow.

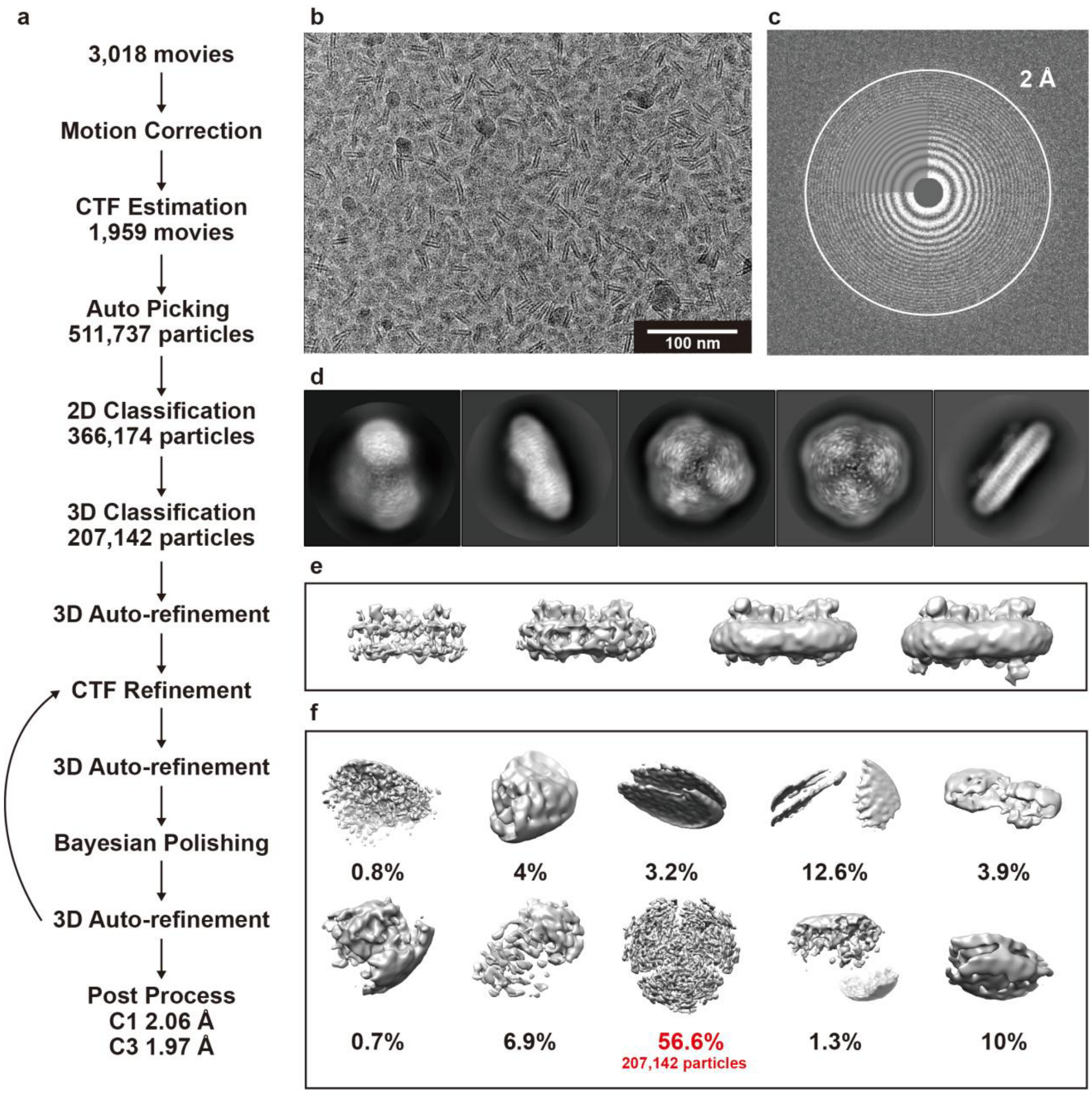

Supplementary Figure 2. Workflow and results of single particle cryo image processing.

a The workflow of data processing was performed in RELION 3.1.

b A representative micrograph.

c Fourier transform with Thon rings extending to 2.0 Å resolution.

d The 2D classes selected for further 3D classification.

e Initial *de novo* model based on auto-picked particles rendered at four different thresholds.

f The results of 3D classification. Particles in the best class (colored in red) were used for 3D refinement.

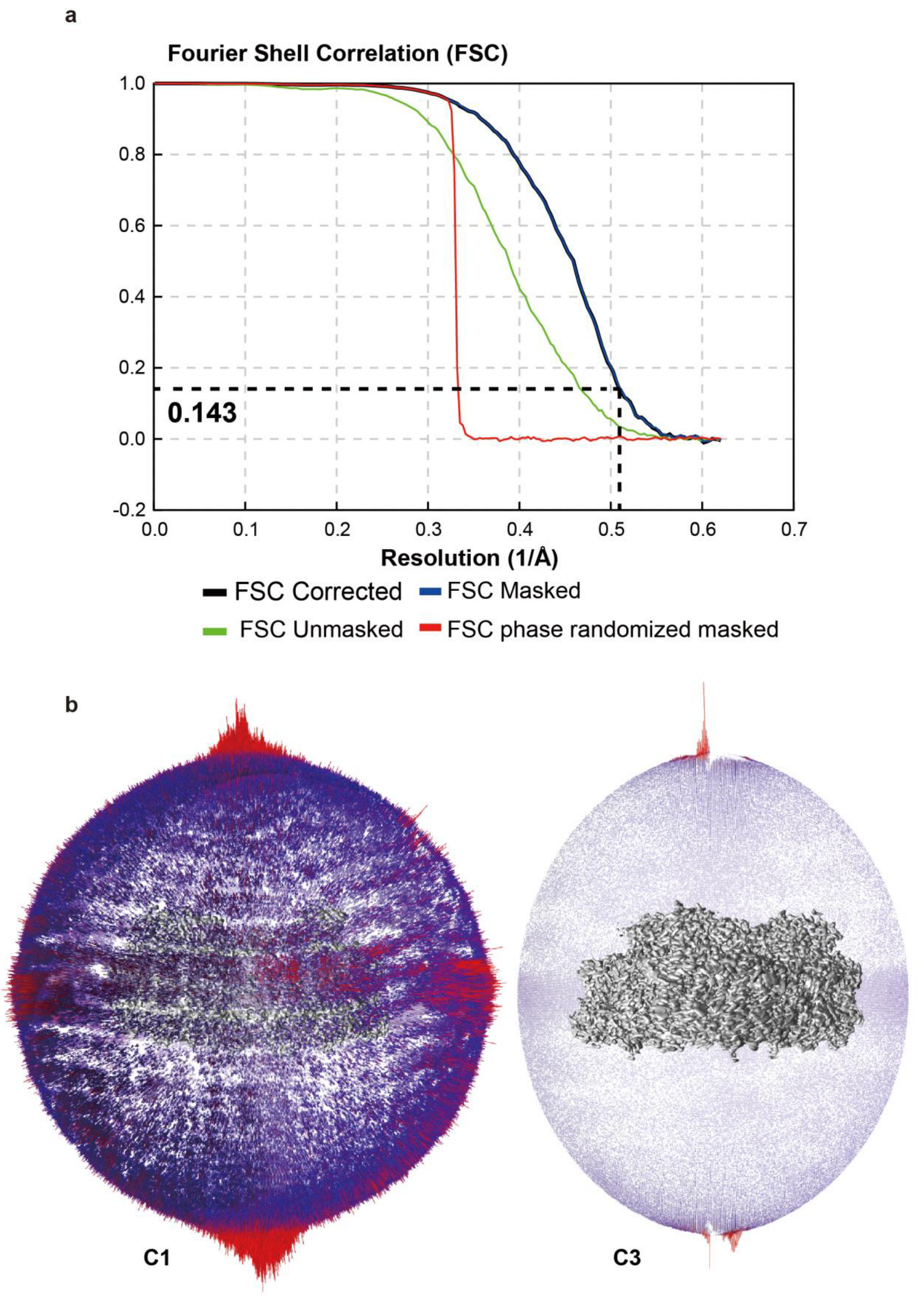

Supplementary Figure 3. The FSC curves and Euler angle distribution.

a The Gold Standard Fourier Shell Correlation (FSC) curves as implemented in Relion 3.1 for the post-processing results of the final map in C3 symmetry. The overall resolution of the masked density map was determined as 1.97 Å at 0.143 Gold Standard FSC cut-off.

b The Euler angle distribution of the final cryo-EM maps in C1 (left) and C3 (right) symmetry.

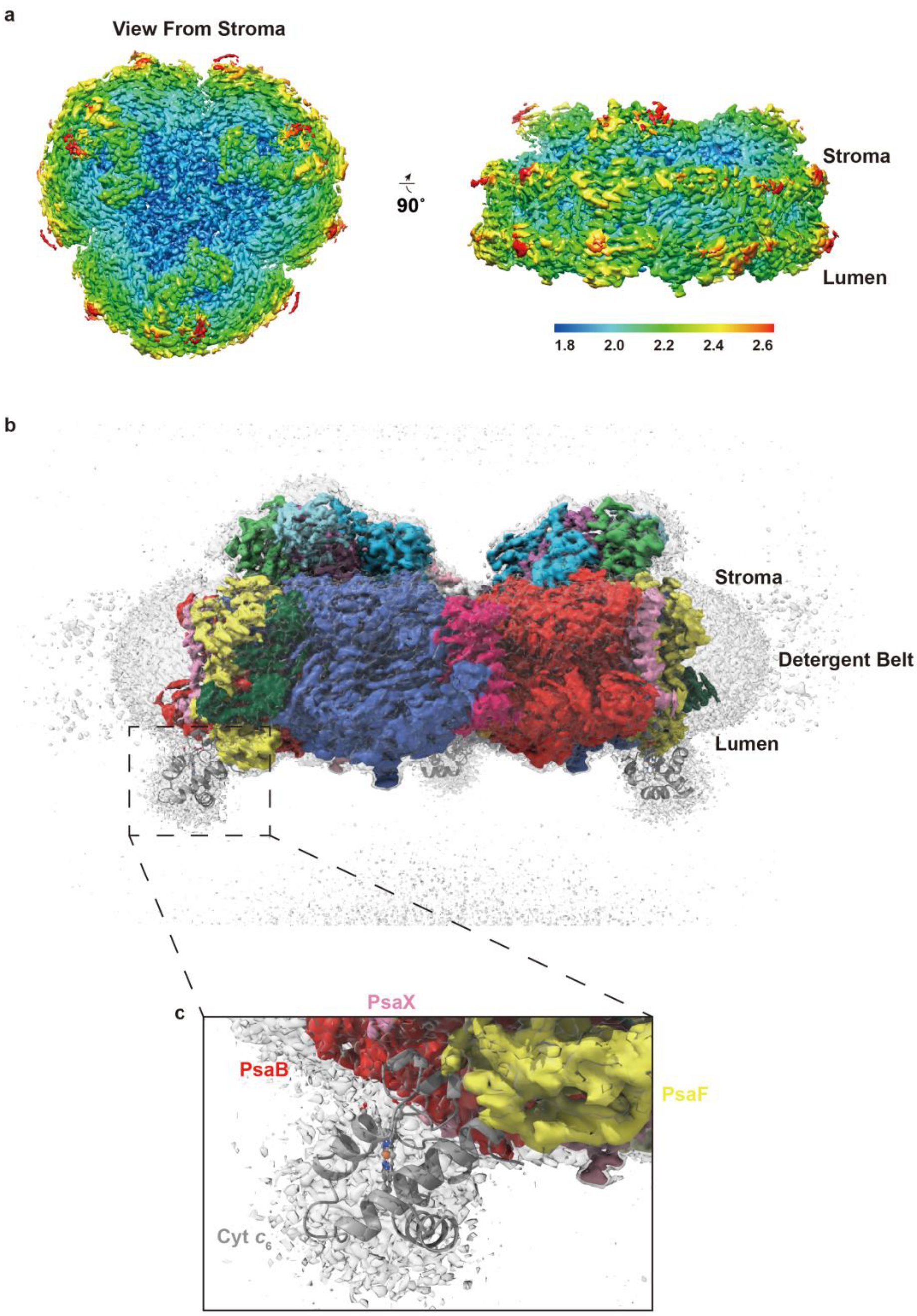

Supplementary Figure 4. Local resolution of the cryo-EM density map and luminal extra density assigned to bound Cyt c_6

a The density map is colored according to the local resolution at a threshold level of 0.007. View from the stromal side (left) and along the membrane plane (right)

b The density map assigned to Cyt c_6 at a lower threshold level. The map is presented in a solid density and colored according to subunits (as defined in Fig. 1) at a threshold level of 0.007 and at a lower threshold level of 0.002 in half-transparent mode. **c.** Close-up of density assigned to Cyt c_6 with the fitted model of the *T. elongatus* Cyt c_6 X-ray crystal structure (PDB ID: 6TR1) shown as a grey ribbon.

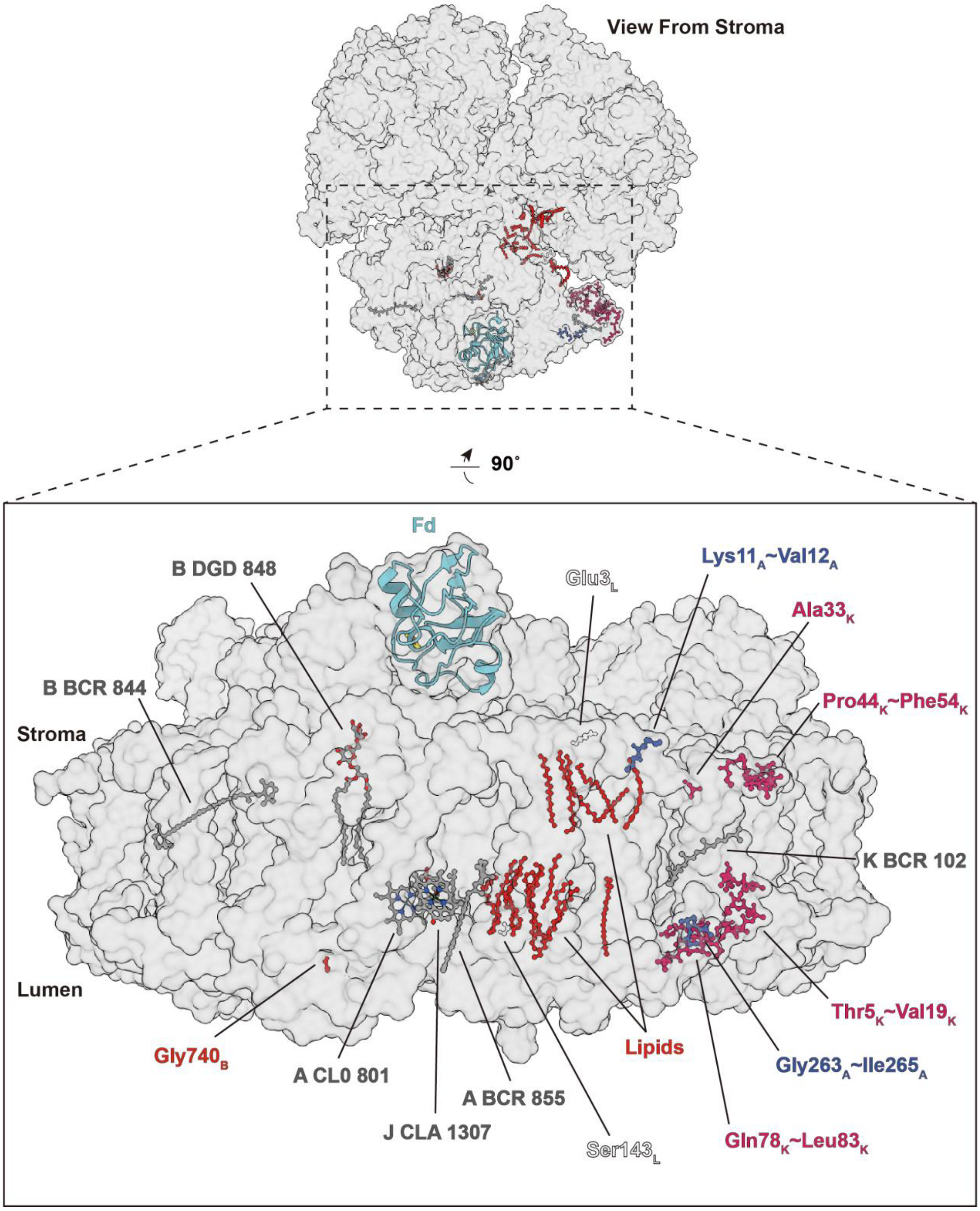

Supplementary Figure 5. Summary of all newly added and modified residues/ligands as presented for one PSI protomer. Additionally, bound Fd is presented in ribbon cartoon and transparent surface. Amino acid residues are colored according to subunits as in Figure 1. Ligands are colored by elements and lipids are shown in red.

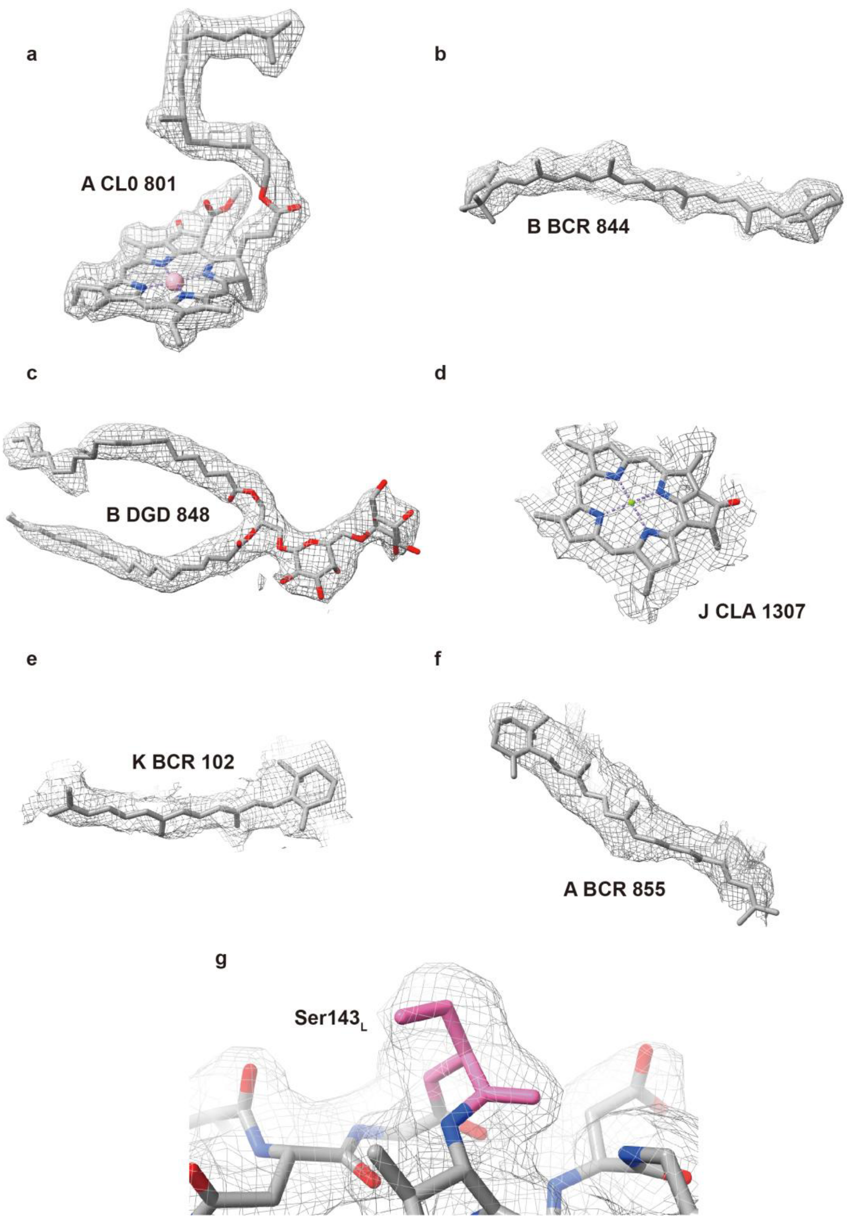

Supplementary Figure 6. Representative additions and modifications to the starting model 1JB0 and their corresponding density maps.

a One chlorophyll of the P700 chlorophyll pair is assigned as the chlorophyll *a'* isomer (PDB Ligands category CL0).

b β-carotene B844 was completely built with all atoms.

c Previously assigned as 1,2-distearoyl-monogalactosyl-diglyceride (MGDG, PDB Ligands category LMG) B848 was modified to digalactosyldiacylglycerol (DGDG, PDB Ligands category DGD) based on the newly detected extra density in the headgroup region.

d Newly assigned chlorophyll J1307 and corresponding density.

e, f Newly assigned, partly built β-carotene K102 and A855, respectively.

g The 143th amino acid residue (colored in pink) in PSI subunit PsaL was modified from leucine to serine based on the Uniprot sequencing result and corresponding density map.

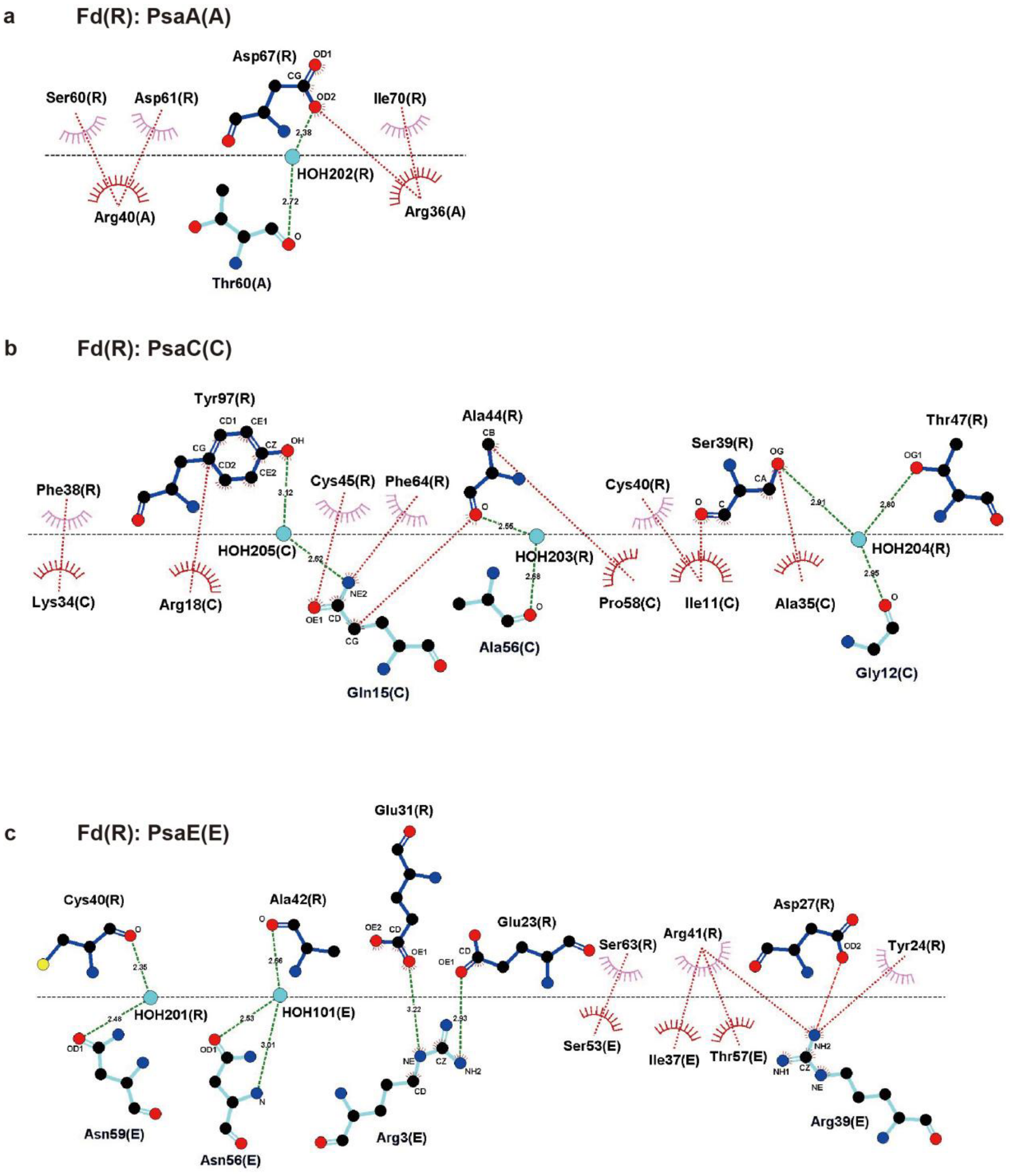

Supplementary Figure 7. Protein-protein interface diagrams of a. Fd:PsaA, b. Fd:PsaC and c. Fd:PsaE derived from DIMPLOT of the LigPlot^{+} suite. Green dashed lines represent potential hydrogen bonds. Red dashed lines show potential non-bonded contacts such as hydrophobic or cation-π interactions.

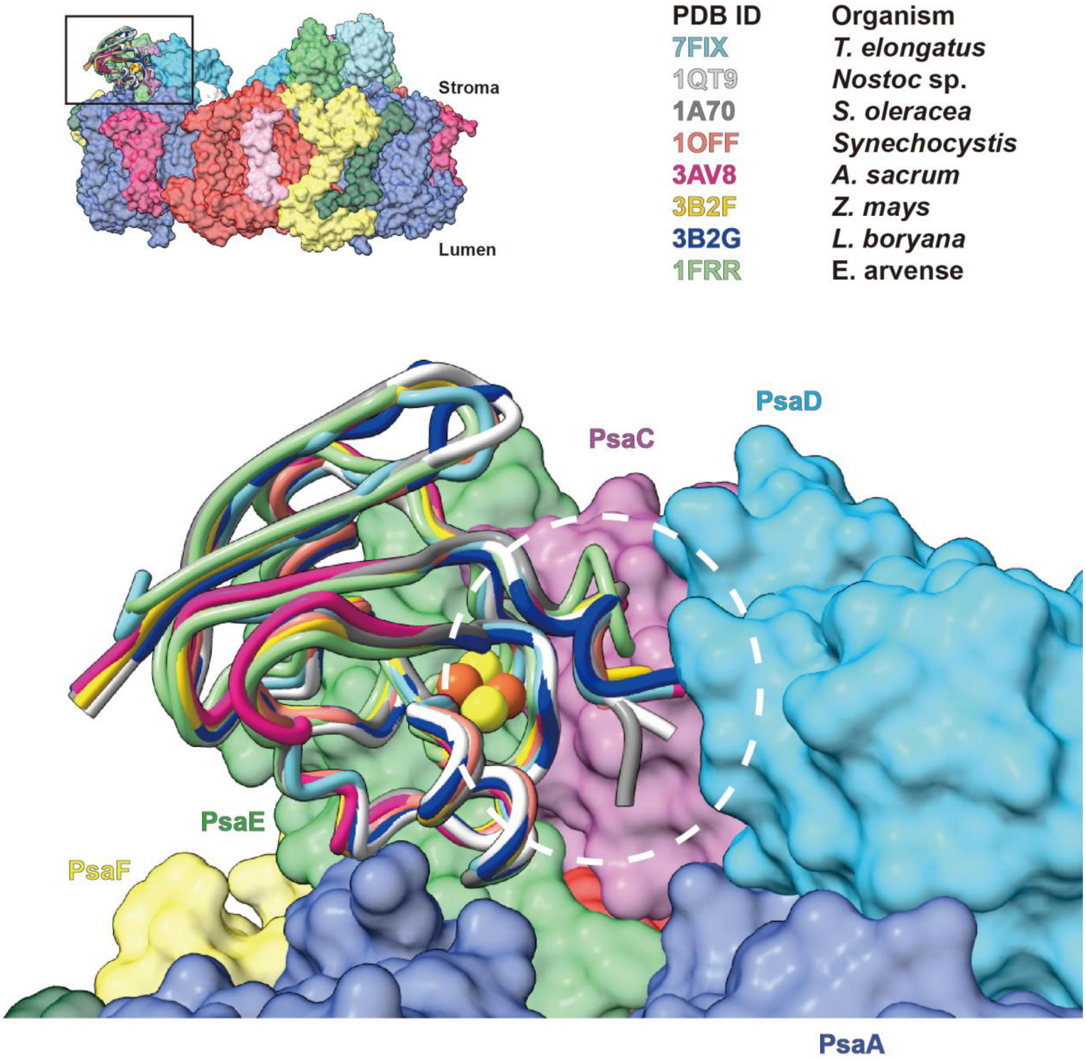

Supplementary Figure 8. Superposition of Ferredoxin structures from diverse sources illustrates the structural flexibility of the C-terminus (white dashed circle).

Supplementary Table 1. Cryo-EM data collection, refinement, and validation statistics

	PSI:Fd complex (EMDB-31605) (PDB 7FIX)
**Data collection and processing**	
Magnification	×60,000
Voltage (kV)	300 kV
Electron exposure (e–/Å^2)	1 / 48
Defocus range (μm)	0.5-1.5
Pixel size (Å)	0.806
Symmetry imposed	C1 / C3
Initial particle images (no.)	367,967
Final particle images (no.)	207,142
Map resolution (Å)	2.06 (C1) / 1.97 (C3)
FSC threshold	0.143
**Refinement**	
Initial model used (PDB code)	1JB0 (PSI), 5AUI (Fd)
Model resolution (Å)	2.06 (C1) / 1.97 (C3)
FSC threshold	0.143
Map sharpening *B* factor (Å^2)	-41.8359 (C1) / -48.4912 (C3)
Model composition	
Non-hydrogen atoms	73929
Protein residues	7137
Ligands	444
R.m.s. deviations	
Bond lengths (Å)	0.010
Bond angles (°)	1.070
Validation	
MolProbity score	1.34
Clashscore	6
Poor rotamers (%)	0
Rama distribution Z-score	1.60 ± 0.10
Ramachandran plot	
Favored (%)	97.92
Allowed (%)	100
Disallowed (%)	0

Supplementary Table 2. A summary of site directed mutation studies on the interaction of PSI and Fd. The residues colored in red were identified as being involved in the interaction with PSI in this study.

Author	Publish year	Protein	Organism	Mutation site (Corresponding residue in 7FIX)	Method	Potential influence
Jonathan Hanley *et al*[1]	1996	PSI PsaD	*Synechocystis* 6803	H97 (H95), K106 (K104), R111 (R109)	Flash-absorption spectroscopy	Fd binding affinity with PSI
Nicolas Fischer *et al*[2]	1998	PSI PsaC	*C. reinhardtii*	**K35 (K34)**	Flash-absorption spectroscopy Electron paramagnetic resonance spectroscopy	Fd binding affinity with PSI
Tetsuyuki Akashi *et al*[3]	1999	Fd	*Z. mays*	E93	Cyclic voltammetry	Fd redox potential
Tetsuyuki Akashi *et al*[3]	1999	Fd	*Z. mays*	D66, **D67**	Affinity chromatography	Fd interaction with FNR and SiR
Nicolas Fischer *et al*[4]	1999	PSI PsaC	*C. reinhardtii*	D9 (D8), **I12 (I11),** T15 (T14), **Q16 (Q15)**	Flash-absorption spectroscopy Electron paramagnetic resonance spectroscopy	Fd binding affinity with PSI
Patrick Barth *et al*[5]	2000	PSI PsaE	*Synechocystis* 6803	**R39**	Flash-absorption spectroscopy	Fd binding affinity with PSI
Bernard Lagoutte *et al*[6]	2001	PSI PsaD	*Synechocystis* 6803	R111 (R109)	NADP+ Photo-reduction Assay Flash-absorption spectroscopy	Fd binding affinity with PSI
Herve Bottin *et al*[7]	2001	PSI PsaD	*Synechocystis* 6803	D100 (D98), E105 (E103), E109 (K107)	Flash-absorption spectroscopy	Fd binding affinity with PSI
Pierre Setif *et al*[8]	2002	PSI PsaC	*T. elongatus*	**K34,** G36	Flash-absorption spectroscopy	Fd binding affinity with PSI
Hisako Kubota-Kawai *et al*[9]	2018	Fd	*T. elongatus*	**Y24, E31, D61, D67,** E71, Y81, E93, **Y97**	Flash-absorption spectroscopy	Fd binding affinity with PSI

Supplementary References

1. Hanley, J. *et al.* Mutagenesis of photosystem I in the region of the ferredoxin cross-linking site: modifications of positively charged amino acids. *Biochemistry* **35**, 8563–8571 (1996).
2. Fischer, N. *et al.* The PsaC subunit of photosystem I provides an essential lysine residue for fast electron transfer to ferredoxin. *The EMBO Journal* **17**, 849–858 (1998).
3. Akashi, T. *et al.* Comparison of the electrostatic binding sites on the surface of ferredoxin for two ferredoxin-dependent enzymes, ferredoxin-$NADP^+$ reductase and sulfite reductase. *Journal of Biological Chemistry* **274**, 29399–29405 (1999).
4. Fischer, N. *et al.* Site-directed mutagenesis of the PsaC subunit of photosystem I. *Journal of Biological Chemistry* **274**, 23333–23340 (1999).
5. Barth, P. *et al.* Essential role of a single arginine of photosystem I in stabilizing the electron transfer complex with ferredoxin. *Journal of Biological Chemistry* **275**, 7030–7036 (2000).
6. Lagoutte, B. *et al.* Multiple functions for the C terminus of the PsaD subunit in the cyanobacterial photosystem I complex. *Plant Physiology* **126**, 307–316 (2001).
7. Bottin, H. *et al.* Role of acidic amino acid residues of PsaD subunit on limiting the affinity of photosystem I for ferredoxin. *Biochemical and Biophysical Research Communications* **287**, 833–836 (2001).
8. Sétif, P. *et al.* The ferredoxin docking site of photosystem I. *Biochimica et Biophysica Acta (BBA) - Bioenergetics* **1555**, 204–209 (2002).
9. Kubota-Kawai, H. *et al.* X-ray structure of an asymmetrical trimeric ferredoxin–photosystem I complex. *Nature Plants* **4**, 218–224 (2018).
