# Supplementary material for: Structure of cyanobacterial photosystem I complexed with ferredoxin at 1.97 Å resolution
Source: Commun Biol. 2022 Sep 12;5:951. doi: 10.1038/s42003-022-03926-4 (PMC9467995; doi:10.1038/s42003-022-03926-4)
Supplement: Supplementary file 3 — Description of Additional Supplementary Files [file 42003_2022_3926_MOESM3_ESM.pdf]

Description of Additional Supplementary Files

File name: Supplementary Data 1
Description: The source data of ITC measurement described in the paper.
