# Supplementary material for: Structure of cyanobacterial photosystem I complexed with ferredoxin at 1.97 Å resolution
Source: Commun Biol. 2022 Sep 12;5:951. doi: 10.1038/s42003-022-03926-4 (PMC9467995; doi:10.1038/s42003-022-03926-4)
Supplement: Supplementary file 5 — Reporting Summary [file 42003_2022_3926_MOESM5_ESM.pdf]

nature portfolio

Corresponding author(s): Christoph Gerle, Genji Kurisu

Last updated by author(s): Jul 20, 2022

Reporting Summary

Nature Portfolio wishes to improve the reproducibility of the work that we publish. This form provides structure for consistency and transparency in reporting. For further information on Nature Portfolio policies, see our Editorial Policies and the Editorial Policy Checklist.

Statistics

For all statistical analyses, confirm that the following items are present in the figure legend, table legend, main text, or Methods section.

n/a	Confirmed	
☒	☐	The exact sample size (n) for each experimental group/condition, given as a discrete number and unit of measurement
☒	☐	A statement on whether measurements were taken from distinct samples or whether the same sample was measured repeatedly
☒	☐	The statistical test(s) used AND whether they are one- or two-sided<br>*Only common tests should be described solely by name; describe more complex techniques in the Methods section.*
☒	☐	A description of all covariates tested
☒	☐	A description of any assumptions or corrections, such as tests of normality and adjustment for multiple comparisons
☒	☐	A full description of the statistical parameters including central tendency (e.g. means) or other basic estimates (e.g. regression coefficient) AND variation (e.g. standard deviation) or associated estimates of uncertainty (e.g. confidence intervals)
☒	☐	For null hypothesis testing, the test statistic (e.g. F, t, r) with confidence intervals, effect sizes, degrees of freedom and P value noted<br>*Give P values as exact values whenever suitable.*
☒	☐	For Bayesian analysis, information on the choice of priors and Markov chain Monte Carlo settings
☒	☐	For hierarchical and complex designs, identification of the appropriate level for tests and full reporting of outcomes
☒	☐	Estimates of effect sizes (e.g. Cohen's d, Pearson's r), indicating how they were calculated

Our web collection on statistics for biologists contains articles on many of the points above.

Software and code

Policy information about availability of computer code

Data collection	Image acquisition was performed using flood beam parallel illumination in bright field imaging mode. Movies were automatically recorded by SerialEM using image shift (5x5 holes per stage position) and a K3 Direct Detection Camera (Gatan, AMETEK, Pleasanton, CA, USA) in CDS mode at a nominal magnification of 60,000× at the camera level, corresponding to a pixel size of 0.806 Å with 48 frames in 3 s exposure time, resulting in a total dose of 48 e−/Å2. A total of 3,018 movies were collected in series within a defocus range of -0.5 µm to -1.5 µm.
Data analysis	COOT (CCP4): manual model building<br>MotionCor2: motion correction of movie frames<br>CTFFIND4: estimation of contrast transfer function<br>RELION 3.1: image processing for 3D reconstruction<br>UCSF Chimera 1.13.1: mask and image preparation, visualization<br>UCSF ChimeraX 1.2.5: map/model visualization<br>Phenix 1.19.2: real space refinement<br>MolProbity: model validation<br>Grade Server: ligand restraints<br>PyMOL 2.3.2: model visualization

For manuscripts utilizing custom algorithms or software that are central to the research but not yet described in published literature, software must be made available to editors and reviewers. We strongly encourage code deposition in a community repository (e.g. GitHub). See the Nature Portfolio guidelines for submitting code & software for further information.

Data

Policy information about availability of data

All manuscripts must include a data availability statement. This statement should provide the following information, where applicable:

- Accession codes, unique identifiers, or web links for publicly available datasets
- A description of any restrictions on data availability
- For clinical datasets or third party data, please ensure that the statement adheres to our policy

All 3,018 raw cryo-EM movies were deposited in the Electron Microscopy Public Image Archive (EMPIAR) under the accession number EMPIAR-10928. The final cryo-EM density map has been deposited in the Electron Microscopy Data Bank (EMDB) under the accession number EMD-31605. The atomic coordinates of the refined PSI:Fd model have been deposited in the Protein Data Bank (PDB) under the accession number 7FIX.

Human research participants

Policy information about studies involving human research participants and Sex and Gender in Research.

Reporting on sex and gender	This study includes no experiment on live vertebrates and/or higher invertebrates.
Population characteristics	*Describe the covariate-relevant population characteristics of the human research participants (e.g. age, genotypic information, past and current diagnosis and treatment categories). If you filled out the behavioural & social sciences study design questions and have nothing to add here, write "See above."*
Recruitment	*Describe how participants were recruited. Outline any potential self-selection bias or other biases that may be present and how these are likely to impact results.*
Ethics oversight	*Identify the organization(s) that approved the study protocol.*

Note that full information on the approval of the study protocol must also be provided in the manuscript.

Field-specific reporting

Please select the one below that is the best fit for your research. If you are not sure, read the appropriate sections before making your selection.

☒ Life sciences ☐ Behavioural & social sciences ☐ Ecological, evolutionary & environmental sciences

For a reference copy of the document with all sections, see nature.com/documents/nr-reporting-summary-flat.pdf

Life sciences study design

All studies must disclose on these points even when the disclosure is negative.

Sample size	The cryo-EM density map was built by 207,142 particles derived from 3,018 cryo-EM movies.
Data exclusions	Following conventional practices of cryo-EM data processing, a significant number of particles with low s/n ratio were excluded as described in the method section.
Replication	The purification of all protein samples were at least repeated twice for checking the performance of purification strategy. Three independent ITC experiments were performed. The cryo-EM data processing was repeated twice (from particle auto-picking to post-processing) and the optimal density map (1.97 Å in C3 symmetry) was used for modeling.
Randomization	Random splitting of image data sets for the Fourier Shell Correlation based resolution estimation was performed.
Blinding	The investigators were not blinded in any ways to identify the sample itself, however, individual particle images were automatically handled in the absence of identification by the investigators.

Reporting for specific materials, systems and methods

We require information from authors about some types of materials, experimental systems and methods used in many studies. Here, indicate whether each material, system or method listed is relevant to your study. If you are not sure if a list item applies to your research, read the appropriate section before selecting a response.

Materials & experimental systems

n/a	Involved in the study
☒	☐ Antibodies
☒	☐ Eukaryotic cell lines
☒	☐ Palaeontology and archaeology
☒	☐ Animals and other organisms
☒	☐ Clinical data
☒	☐ Dual use research of concern

Methods

n/a	Involved in the study
☒	☐ ChIP-seq
☒	☐ Flow cytometry
☒	☐ MRI-based neuroimaging
